# PDGF-Rα gene expression predicts proliferation, but PDGF-A suppresses transdifferentiation of neonatal mouse lung myofibroblasts

**DOI:** 10.1186/1465-9921-10-119

**Published:** 2009-11-25

**Authors:** Patricia W Kimani, Amey J Holmes, Ruth E Grossmann, Stephen E McGowan

**Affiliations:** 1 Molecular and Cellular Biology Ph.D. program, University of Iowa, Iowa City, Iowa, USA; 2 Research Service, Iowa City Department of Veterans Affairs Medical Center, University of Iowa Carver College of Medicine, Iowa City, Iowa, USA; 3 Emory University, Graduate division of Biological and Biomedical sciences, Atlanta, Georgia, USA

## Abstract

**Background:**

Platelet-derived growth factor A (PDGF-A) signals solely through PDGF-Rα, and is required for fibroblast proliferation and transdifferentiation (fibroblast to myofibroblast conversion) during alveolar development, because *pdgfa*-null mice lack both myofibroblasts and alveoli. However, these PDGF-A-mediated mechanisms remain incompletely defined. At postnatal days 4 and 12 (P4 and P12), using mouse lung fibroblasts, we examined (a) how PDGF-Rα correlates with ki67 (proliferation marker) or alpha-smooth muscle actin (αSMA, myofibroblast marker) expression, and (b) whether PDGF-A directly affects αSMA or modifies stimulation by transforming growth factor beta (TGFβ).

**Methods:**

Using flow cytometry we examined PDGF-Rα, αSMA and Ki67 in mice which express green fluorescent protein (GFP) as a marker for PDGF-Rα expression. Using real-time RT-PCR we quantified αSMA mRNA in cultured Mlg neonatal mouse lung fibroblasts after treatment with PDGF-A, and/or TGFβ.

**Results:**

The intensity of GFP-fluorescence enabled us to distinguish three groups of fibroblasts which exhibited absent, lower, or higher levels of PDGF-Rα. At P4, more of the higher than lower PDGF-Rα + fibroblasts contained Ki67 (Ki67+), and Ki67+ fibroblasts predominated in the αSMA + but not the αSMA- population. By P12, Ki67+ fibroblasts comprised a minority in both the PDGF-Rα + and αSMA+ populations. At P4, most Ki67+ fibroblasts were PDGF-Rα + and αSMA- whereas at P12, most Ki67+ fibroblasts were PDGF-Rα- and αSMA-. More of the PDGF-Rα + than - fibroblasts contained αSMA at both P4 and P12. In the lung, proximate αSMA was more abundant around nuclei in cells expressing high than low levels of PDGF-Rα at both P4 and P12. Nuclear SMAD 2/3 declined from P4 to P12 in PDGF-Rα-, but not in PDGF-Rα + cells. In Mlg fibroblasts, αSMA mRNA increased after exposure to TGFβ, but declined after treatment with PDGF-A.

**Conclusion:**

During both septal eruption (P4) and elongation (P12), alveolar PDGF-Rα may enhance the propensity of fibroblasts to transdifferentiate rather than directly stimulate αSMA, which preferentially localizes to non-proliferating fibroblasts. In accordance, PDGF-Rα more dominantly influences fibroblast proliferation at P4 than at P12. In the lung, TGFβ may overshadow the antagonistic effects of PDGF-A/PDGF-Rα signaling, enhancing αSMA-abundance in PDGF-Rα-expressing fibroblasts.

## Background

Fibroblast proliferation and transdifferentiation (fibroblast to myofibroblast conversion) are key cellular functions required for both normal alveolar development and the progression of fibrotic pulmonary diseases. Whereas many studies have focused on disease states, similar mechanisms may account for both normal lung development and remodelling after lung injury. Increased knowledge of the links between these processes could introduce more effective treatment strategies for terminal diseases such as intrapulmonary fibrosis and chronic obstructive pulmonary disease [1-3].

Alveolar septal formation involves coordinated expansion of epithelial and mesenchymal cells and establishment of a specialized epithelial-endothelial interface that permits efficient gas exchange. The attenuated alveolar-capillary basement membrane is interrupted at the thickened portions of septum, which reside primarily at the base and at the alveolar entry rings. Differentiated mesenchymal cells such as the myofibroblasts, reside in these regions and deposit extracellular collagen and elastic fibers that support the delicate intervening alveolar-capillary interface. Myofibroblasts have been identified in the developing pulmonary alveolar interstitium of a variety of species, including rats [4], mice [5], sheep [6] and humans [7]. These specialized fibroblasts display smooth muscle cell-like features, such as alpha-smooth muscle actin (αSMA) expression [8,9].

The platelet-derived growth factor (PDGF) family, consisting of the ligands A, B, C and D and the receptor tyrosine kinases PDGF-Rα and β, regulate the proliferation, migration and differentiation of mesenchymal cells during embryonic and postnatal development [10]. All four ligands form dimers; whereas A and B can both homo- and heterodimerize (AA, AB or BB), C and D exist solely as homodimers (CC and DD). PDGF A signals solely via PDGF-Rα, B and C via both PDGF-Rα and β, and D solely via PDGF-Rβ. PDGFs A, B and C are expressed in the developing mouse lung[11,12], but mice that are null for the *pdgf-a *gene[13,14] display reduced numbers of PDGF-Rα-expressing cells, lack myofibroblasts and elastin deposits, and fail to develop alveoli. A role for PDGFs B and C in alveolar septal formation and myofibroblast development has not been identified, as both *pdgfs-b *and *-c *null mice die *in utero *[15,16]. Therefore, the behavior of PDGF-Rα-expressing mesenchymal cells may necessarily, but not exclusively reflect the actions of PDGF-A.

Whereas it is well known that fibroblasts actively proliferate and deposit alveolar septal ECM [17], and that PDGF-A/PDGF-Rα signaling is required for myofibroblast differentiation [13,14], it remains unclear how PDGF-Rα expression influences the timing of fibroblast proliferation and differentiation. Stimulatory and inhibitory signals normally confine myofibroblasts to the period of pulmonary alveolarization and myofibroblasts are rarely observed in the adult lung [18]. Understanding more about these normal regulatory signals is important, because myofibroblasts reappear in injured and inflamed lung tissue, and contribute to the progression of fibrosis which leads to scarring and eventual loss of lung function [19].

Using green fluorescent protein (GFP) as an *in vivo *marker for pulmonary PDGF-Rα expression, we have demonstrated that fibroblasts actively expressing the PDGF-Rα gene are more likely to contain αSMA (characteristic of myofibroblasts) than their non-expressing counterparts [20]. We have now investigated the relationships between proliferation (using ki67 as a marker for proliferation) and αSMA gene expression in individual PDGF-Rα-expressing + and non-expressing - lung fibroblasts.

Because PDGF-B antagonizes αSMA gene expression in smooth muscle cells [21], we hypothesized that PDGF-Rα-expressing lung fibroblasts differ from non-expressing lung fibroblasts in their response to transforming growth factor beta (TGFβ). TGFβ1 is well characterized as a mediator of fibroblast to myofibroblast conversion [22-24] and is important for alveolar development [25-27]. Therefore using cultured mouse lung fibroblasts, we have examined potential direct effects of PDGF-A on αSMA. We have also investigated whether the nuclear abundance of the TGFβ signalling complex, SMAD 2/3 [28,29], differs in PDGF-Rα expressing and non-expressing lung fibroblasts.

## Methods

### Reagents

Collagenase type I (288 U/mg), trypsin (8500 U/mg), DNAse I (2080 KU/mg) and calcium chloride were purchased from Sigma Aldrich (St Louis, MO). Monoclonal rat anti-ki67 primary antibody was purchased from DAKO North America, Inc. (Carpenteria, CA). Superscipt II reverse transcriptase, dNTPs, Quant IT™ Oligreen R ssDNA assay kit, Rat IgG2a isotype control, popo 3 iodide dimeric cyanine nucleic acid stain (C_4_H_58_I_4_N_6_O_2_), and Alexa Fluor 647-goat anti-rat and -goat anti-rabbit secondary IgGs were purchased from Invitrogen (Carlsbad, CA). Phycoerythrin (PE) - anti-human monoclonal α-smooth muscle actin antibody, Mouse IgG2a PE isotype control, recombinant human PDGF-AA (221-AA) and porcine TGFβ1 (101-B1) were purchased from R & D Systems (Minneapolis, MN). BD Compbeads compensation particle sets for optimising the flow cytometry fluorescence signals were purchased from BD Biosciences. Rabbit polyclonal anti-SMAD 2/3 primary antibody was purchased from Santa Cruz Biotechnology (Santa Cruz, CA). RNAse H was purchased from New England Laboratories (Ipswich, MA). NucAway Spin columns and Taqman Gene Expression Assays for α-smooth muscle actin and β-2-microglobulin were purchased from Applied Biosystems.

### Animals

Mice bearing the PDGF-Rα-GFP allele were originally obtained from Dr. Philippe Soriano (Fred Hutchinson Cancer Center, Seattle, WA), and details regarding their characterization and phenotype have been reported elsewhere [30,31]. Briefly, the human histone *H2B *gene was subcloned into a pEGFPN1 cloning vector to generate the H2B-GFP construct encoding a nuclear form of GFP [32]. The resultant H2B-GFP construct was inserted into targeting (knock in) vectors. By homologous recombination in the mouse embryos, these vectors replaced a fragment in the wildtype *pdgf-rα *allele corresponding to the signal peptide and the first two immunoglobulin domains. Thus nuclear GFP expression in the transgenic mice is under the control of the endogenous *pdgf-rα *promoter. Soriano's group observed that GFP expression in the PDGF-Rα-GFP mice recapitulated endogenous spatial and temporal PDGF-Rα expression [30]. We used mice that were heterozygous for the *pdgf-rα-gfp *allele; thus the mice still carry one functional *pdgf-rα *allele. The heterozygous mice phenotypically resemble wildtype (GFP^-^) mice [30]. Mice were housed in Thoren cages with access to food and water *ad libitum *in a thermally regulated environment and a 12-hour light/dark cycle. Protocols for animal use were approved by the Iowa City Veterans Affairs Medical Center animal use committee.

### Isolation of mouse lung fibroblasts

Prior to the fibroblast isolation, mice were screened for the PDGF-Rα-GFP allele using either PCR or fluorescence microscopy. For PCR, 1 μl of tail DNA was mixed with dNTPs, MgCl_2_, 10× PCR buffer, and the following GFP primers: EGFP forward - 5' - GCC ATG CCC GAA GGC TAC GTC - 3' EGFP reverse - 5' - GGG TGC TCA GGT AGT GGT TGT C - 3' The amplification reaction was performed in a thermocycler unit (Techne Limited, Cambridge UK). To screen the animals using fluorescence microscopy, a small piece of the lung was compressed on a glass slide using a coverslip, and the presence or absence of GFP fluorescence was observed using an epifluorescence microscope.

Primary mouse lung fibroblasts were isolated from 4 and 12-day old mice using a previously reported method, with minor modifications [33]. After the animals had been anesthetized with 25 μl of a mixture of 90 mg/ml ketamine and 10 mg/ml xylazine, their lungs were removed, pooled together and digested for a total period of 1 h, with removal of the dispersed cells every 10 minutes. The enzyme solution consisted of Hanks buffered salt solution (HBSS, without Ca or Mg) with 300 μg/ml collagenase type I, 250 U trypsin (8500 U/mg solid), 75 μg/ml DNAse I and 5 mM calcium chloride. After each digestion interval, the solution containing dispersed cells was passed through 100 μm mesh into Ham's F-12 k media containing 10% FBS, and the undigested lung tissue placed in fresh enzyme solution. Once digestion was complete (after 3-4 digestions), erythrocytes were separated from the fibroblasts by sedimentation through a 40% Percoll solution. To select for fibroblasts, cells were adhered to 100 mm tissue culture dishes for 1 h at 37°C. Adherent cells were harvested by trypsinization followed by gentle scraping and centrifugation.

### Preparation of freshly isolated mouse lung fibroblasts for Flow Cytometry analysis of αSMA and Ki67

Freshly isolated mouse lung fibroblasts were proportioned into aliquots of 5 × 10^5 ^cells and washed several times by mixing with 1× phosphate-buffered saline (PBS - 0.145 M NaCl, 0.0015 M KH_2_PO_4_, 0.0027 M KCl, 0.0086 M Na_2_HPO_4_, pH 7.4) followed by centrifugation. Following rinsing, the fibroblasts were fixed in 2% paraformaldehyde/PBS (v/v) for 20 min with shaking at 4°C. After additional rinses in PBS, the fibroblasts were permeabilized in 0.1% (w/v) saponin in PBS at 4°C for 10 min. After the permeabilization step, non-specific binding was reduced by incubating the fibroblasts in 5% non-fat dry milk/0.1% saponin/PBS for 2 h at 4°C. Following the blocking step, monoclonal rat anti-ki67 primary antibody (1:300 dilution, clone TEC-3) or rat IgG2a was added to the fibroblasts, and the cells incubated overnight at 4°C. The next day, the cells were rinsed in 0.1% saponin/PBS. In a sequential manner, Alexa Fluor 647-goat-anti rat secondary IgG (1 μg/ml) and phycoerythrin (PE) - anti-α-smooth muscle actin primary antibody (0.0625 μg/ml) or PE-mouse IgG2a were added and the cells incubated for 1 h at 4°C. Finally, the cells were rinsed in 0.1% saponin/PBS, and resuspended in PBS for flow cytometry.

### Collection and analysis of Flow Cytometry data

Flow cytometry was performed at the University of Iowa Flow Cytometry facility using the LSR II instrument (BD Bioscience). An unstained sample was used to gate the forward and side scatter pattern of the cell population of interest. Optimization of signal as well as compensation for overlap between the PE and GFP or the Alexa Fluor 647 signals was achieved using BD Comp beads - polystyrene microparticles coated with mouse IgG_κ _or FBS (negative control, BD Bioscience) - stained in the same manner as the fibroblasts. The resultant data were analyzed using Cell Quest Pro (BD Biosciences), Microsoft Excel and Sigma Stat software (Jandal Scientific, San Rafael, CA). Each "n" represents a cohort of 2 to 5 mice, of the same age, whose lungs had been pooled and digested together. A minimum of 9000 gated events were analyzed for each "n." Background compensation was determined from the IgG controls prior to calculating the proportions of the different fibroblast populations.

### Evaluating the purity of the fibroblast isolate

The fibroblast isolate was evaluated for epithelial, endothelial and macrophage contamination using flow cytometry. Epithelial cells were detected after fixation using monoclonal mouse anti-cytokeratin 18 (Sigma), followed by Alexa Fluor 647-conjugated goat anti-mouse secondary IgG. Anti-cytokeratin independent staining was assessed using only the secondary antibody. Endothelial cells were stained prior to fixation using PE-conjugated monoclonal rat anti-mouse CD31 or -mouse IgG_1 _isotype control (BD Biosciences). Macrophages were detected after fixation and permeabilization using Alexa Fluor 647-conjugated-rat anti-mouse mannose receptor c type 1 (CD206) or -IgG_2a _(Serotec). Proportions of cells staining positive for either cytokeratin 18, CD31 or CD206 were determined after correcting for primary antibody-independent fluorescence.

### Preparation of Lung tissue for Confocal Microscopy studies

Confocal microscopy studies utilized either 100 or 7 μm lung tissue sections obtained from mice at postnatal days 4 and 12. Preparation of the 100 μm sections has been described elsewhere [20]. For the preparation of the 7 μm sections, mice were anesthetized and their chests opened to expose their lungs. The lungs were perfused transcardially with 1× PBS and then inflated to total lung capacity with a uniform quantity (for a particular age) of 75% Optimal Cutting Temperature (OCT) Medium, dissected out of the thoracic cavity, and frozen in a bath of liquid nitrogen and isomethylbutane. The frozen lung tissue was cut into 7 μm sections using a cryostat, mounted on glass slides and stored at -20°C prior to fixation.

### Immunofluorescence and Confocal Microscopy Imaging

The 100 μm sections were stained with Cy3-α-smooth muscle actin monoclonal antibody (R & D systems, Minneapolis, MN) and a lipid counterstain, 1,1'-dioctadecyl-3,3,3^/^,3^/^-tetramethylindodicarbo-cyanine perchlorate (DiD, Invitrogen, Carlsbad, CA). The staining and imaging processes have already been described in detail [20].

The 7 μm lung tissue sections were equilibrated at 4°C in 1× PBS for 5 min, and then fixed in 2% paraformaldehyde for 1 h at 4°C. Fixed sections were then rinsed extensively with 1× PBS. All subsequent antibody incubations were performed in a humidified chamber, and rinses in Coplin jars. Following the rinses, the lung tissue was permeabilized in 0.1% Triton X-100/3 mg/ml bovine serum albumin (BSA) (permeabilization buffer) for 30 min at RT. To minimize non-specific antibody binding, the tissue was then placed in blocking solution - permeabilization buffer/5% normal goat serum - overnight at 4°C. After the blocking step, rabbit polyclonal anti-SMAD 2/3 primary antibody (0.5 μg/ml) was added to the blocking solution, and the incubation performed overnight at 4°C. The lung tissue was subsequently rinsed with permeabilization buffer, and then incubated with Alexa Fluor 647-goat anti-rabbit secondary IgG (4 μg/ml) for 1.5 h at RT. After more rinsing, the tissue was incubated for 40 min at 37°C with 1 × 10^-7 ^M popo 3 iodide.

Images of the stained sections were acquired from randomly selected fields using Zeiss LSM510 Laser Scanning Confocal Microscopes (LSCM). The following excitation/emission filters were used: a 488/505-530 nm band pass filter to detect GFP fluorescence, a 533/560 nm long pass filter to detect PoPo 3 iodide fluorescence, a 543/560-615 nm band pass filter to detect Cy3-α-smooth muscle actin antibody, and a 650 nm long pass filter for detecting either DiD or Alexa Fluor 647-goat anti-rabbit secondary IgG. Z-stack images were obtained at 1024 × 1024 pixel density.

### Analysis of PDGF-Rα and αSMA in images acquired by laser scanning confocal microscopy

For analysis of images acquired from the 100 μm sections, alveoli were identified and annotated in the 3 μm interval z-stacks using StereoInvestigator (MBF Bioscience, Williston, VT). Each alveolus selected for analysis was traced through the z-stack, and was defined as having an entry ring and a base [20]. Inspection of the tissues revealed that there were different levels of intensity of GFP-fluorescence that were not related to the distance from the laser beam or the detector. Therefore we devised uniform segmentation criteria using IP lab software (BD Bioscience, Rockville, MD), which discriminated between populations of nuclei with high and low intensities of GFP fluorescence. Because the intensity of the GFP was intrinsic to the tissue and not dependent on antibody staining, it is representative of the level of PDGF-Rα gene expression. To examine PDGF-Rα-GFP distribution at the alveolar entry ring and base, "low" and "high" PDGF-Rα-GFP cells were manually counted at each level.

The intensity of α-SMA staining was antibody-dependent, thus we always imaged tissues obtained from P4 and P12 animals that were stained concurrently. To avoid variability related to distances from the laser beam, alveolar entry rings located 1/4 to 1/2 of the way into the z-stack were used for analysis. To quantify the pixel density of αSMA associated with PDGF-Rα expression at the alveolar entry ring, a circle measuring 9 μm in diameter was drawn around each PDGF-Rα-GFP nucleus. The pixel density of αSMA was ascertained relative to this uniform region of interest. The entry ring was defined by staining of αSMA, which occupied at least 75% of the circumferential transition from an alveolar duct into an alveolus. The areal pixel densities of αSMA were expressed in binned segments.

### Analysis of nuclear SMAD 2/3 in the alveolar region

Localization of SMAD 2/3 to nuclei in cells that either expressed PDGF-Rα (nuclei with GFP) or did not express PDGF-Rα (nuclei without GFP) was ascertained as follows: The fluorescent DNA dye, PoPo3, was assigned a blue pseudocolor. In the blue-only image, we optimized the threshold intensity to accurately select pixels representing all blue nuclei (nuclei stained with PoPo3), and quantified the pixel area covered by all the nuclei (all nuclei comprised both those with and without GFP). Next, in the green-only image, we selected only for the nuclei, which also contained GFP above a fixed threshold intensity, and quantified the total pixel area covered by these nuclei. In order to ascertain the nuclear area occupied by SMAD 2/3 (assigned the color red) but not GFP, uniform segmentation criteria were applied to the red-green-blue merged image, enabling selection of pixel segments which contained blue and red, but not green (magenta). In order to ascertain the nuclear area occupied by SMAD 2/3 and GFP, we applied uniform segmentation criteria to the RGB merged image, enabling selection of pixels which contained green and red (yellow). Finally, we ascertained the proportional pixel areas of SMAD 2/3 in nuclei that did or did not contain GFP using the following formulas: (a) Fraction of SMAD 2/3 within GFP^- ^nuclei = area of magenta pixels ÷ (area of all nuclei minus area of GFP^+ ^nuclei) (b) Fraction of SMAD 2/3 within GFP^+ ^nuclei = area of yellow pixels ÷ area of GFP^+ ^nuclei.

We applied this procedure to 3 different mice at P4 and at P12, and examined all of the alveoli in at least 10 different randomly selected fields per animal. The results were expressed as mean ± SD, and ANOVA on ranks followed by Dunn's *posthoc *test was used to determine statistical significance.

### Cell Culture

The neonatal mouse lung fibroblast cell line, MLg 2908 (CCL-206) was obtained from the American Type Culture Collection (Manassas, VA). The cells were maintained at 5% CO_2 _and 37°C in T-75 flasks with MEM media containing 10% FBS (v/v) and sub-cultured every 4 days. For experiments, the cells were seeded into 100 mm tissue culture dishes. Cells were used between passages 3 and 10.

### Assessing the effect of exogenous PDGF-AA and/or TGFβ treatment on αSMA mRNA expression in Mlg Cells

On day 1, the Mlg 2908 cells were plated in 10% FBS/MEM media onto 100 mm tissue culture treated dishes at a density of either 2 × 10^5^(sub-confluent) or 2 × 10^6 ^(confluent) cells. On day 2, the cell monolayer was rinsed with Opti MEM I media, and 0.2% BSA/Opti MEM 1 media was added. The concentrations of PDGF-AA and/or TGFβ indicated in the figure legends were added 24 or 48 h after serum starvation. Total RNA was isolated by the guanidinium chloride method [34] 24 or 48 h post treatment.

### Reverse Transcription of total RNA

Oligo (dT) primers and dNTPs were added to the total RNA, and the RNA was denatured at 70°C for 15 min, and then cooled on ice for 5 min. Subsequently, dithiothreitol (DTT) and 5× first strand buffer were added to the samples, and they were annealed at 42°C for 5 min. To initiate the reverse transcriptase reaction, Superscript II (Invitrogen) was then added, and the samples heated to 42°C for 50 min. The reaction was subsequently quenched by heating the samples to 70°C for 15 min. To remove any residual RNA, the samples were treated with RNAse H at 37°C for 30 min. The cDNA product was diluted with TE buffer, and placed on NucAway Spin Columns to remove salts and unincorporated nucleotides. Concentrations of cDNA were determined using the Quant IT™ Oligreen R ssDNA Assay Kit.

### Real-time quantitative PCR (RT-qPCR)

Quantification of αSMA mRNA was achieved by mixing 5 ng of cDNA with 1.5 μl of Taqman Gene Expression Assay for αSMA (probe number Mm01546133_m1) or β-2-microglobulin (probe number Mm00437762_m1) (housekeeping gene), and 15 μl of Taqman Universal PCR MasterMix (catalogue number 4324018), to a final volume of 20 μl. The probes are designed to amplify exon-intron junctions to minimize amplification of any remaining genomic DNA. The Real-time PCR was performed in triplicate (3 amplification reactions for one reverse-transcribed RNA) using an Applied Biosystems Model 7500 Sequence Detection System. At least four independent experiments were performed. Values for the αSMA expression were normalized to β-2-microglobulin, and calculated in Excel using the 2^(-ΔΔCT) ^relative quantification method [35].

### Statistics

Data were expressed as means ± SD. Unless otherwise indicated, statistical significance was determined by performing a 2-way Analysis of Variance followed by a Student-Newman-Keuls *post-hoc *test. Values of p < 0.05 were considered significant.

## Results

### The abundance of PDGF-Rα-expressing fibroblasts, which display varied PDGF-Rα levels, changes during alveolar development

In order to determine whether the size of the PDGF-Rα-expressing fibroblast subpopulation changed according to the stage of alveolarization, mouse lung fibroblasts were isolated from animals at 4 and 12 days of age (P4 and P12). The purity of the fibroblasts was assessed by staining for cellular markers specific for epithelial (cytokeratin 18), macrophage (CD 206) and endothelial (CD31) cells. At P4, epithelial, macrophage and endothelial comprised 2.7 ± 3% (n = 4), 12.9 ± 3% (n = 3) and 1.0 ± 0.3% (n = 2, mean ± SD) respectively, of the cells which adhered to culture plastic. At P12, epithelial, macrophage and endothelial cells comprised 8.1 ± 6% (n = 2), 18.1 ± 1% (n = 3) and 1.6 ± 0.5% (n = 3, mean ± SD) respectively, of the adherent cells. At both ages, less than 6% of GFP positive cells stained positive for any of these cellular markers.

**Figure 1:**
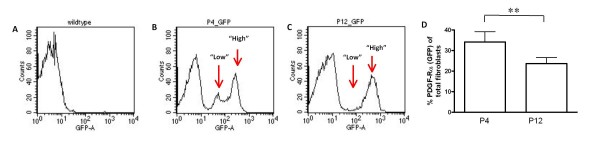
**The pattern of PDGF-Rα expression in mouse lung fibroblasts changes during alveolar development**. Mouse lung fibroblasts were isolated from either wildtype (WT, A) or mice carrying the PDGF-Rα-GFP transgene (GFP, B and C). Expression of PDGF-Rα-GFP was compared at postnatal days 4 (P4, B) and 12 (P12, C). The average abundance of GFP-positive fibroblasts expressed as a percentage of total fibroblasts (cells adherent to plastic after 1 h), is summarized in the column graph (D). For P4, n = 5 groups of 2 to 4 mice, and at P12, n = 7 groups of 2 to 4 mice. For the FACS histograms, "counts" on the y-axis = the number of events, and on the x-axis, "GFP-A," = GFP Area. **, p < 0.001.

Based on the relative intensities of GFP fluorescence as measured on the X-axis of the flow cytometry histogram (Figure 1B and 1C) we observed that the fibroblasts could be distinguished as "low" and "high" PDGF-Rα-expressing groups. In addition, the "low" was distinctively separable from the "high" population at postnatal day 4 (P4) (1 B), but not at P12 (1 C). Furthermore, the overall proportions of PDGF-Rα (GFP)-expressing fibroblasts were lower at P12 compared to P4 (1 D, p < 0.05). These results suggest that PDGF-Rα expression levels in the fibroblasts change as alveolarization progresses.

### There is a positive correlation between fibroblast PDGF-Rα expression levels and proliferation at P4, but not P12

**Figure 2:**
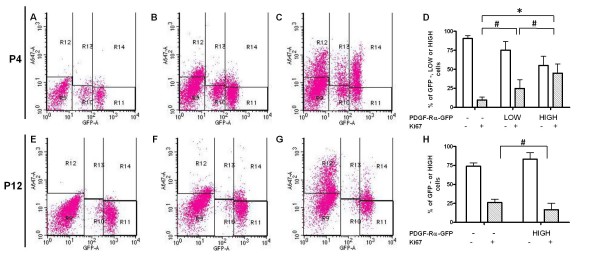
**PDGF-Rα expression positively correlates with proliferation at P4, but not at P12**. Representative FACS histograms of freshly isolated mouse lung fibroblasts from PDGF-Rα-GFP mice aged P4 (A - C) and P12 (E - G). Fibroblasts were stained for ki67 (C and G) or IgG control (B and F), followed by Alexa-Fluor 647-conjugated secondary antibody (A647, y-axis). Negative controls stained with the secondary antibody only are shown in A and E. To analyze the flow cytometry output, cell populations were divided into regions; region 9 is R9, and so on. The proportions of non-expressing, or PDGF-Rα-GFP "low" or "high" subpopulations (shown by GFP fluorescence on the x-axis) that were positive or negative for ki67 were determined. Average percentages were summarized in column graphs for P4 (D) and P12 (H). At P12, only the "high" subpopulation was examined because the "low" subpopulation could not be accurately determined. Non-specific binding determined from the isotype-matched non-immune IgG control samples was removed from each experiment. Means are from n = 5 (groups of 2-4 mice) for P4, and n = 7 for P12. Error bars represent SD; #, P < 0.05 and *, P < 0.001. For the FACS histograms, on the x-axis, "GFP-A" = GFP Area and on the y-axis, "A647-A" = A647 Area.

PDGF-A null mice lack myofibroblasts and secondary alveolar septa, which is thought to result from a failure of *pdgf-rα-*expressing mesenchymal cell precursors to migrate from proximal tubules to the terminal primary septae [13,14]. Because fibroblasts within the developing secondary septae are known to actively proliferate during septal formation, lack of alveolar septa could also result from diminished PDGF-A-mediated fibroblast proliferation. In order to examine the correlation between PDGF-Rα expression levels and the timing of fibroblast proliferation, we used flow cytometry to quantify the proportions of Ki-67-containing, PDGF-Rα-GFP+ and PDGF-Rα-GFP-fibroblasts at P4 (Figure 2D) and P12 (Figure 2H). In the flow cytometry histograms, the "low" and "high" PDGF-Rα subpopulations were distinct at P4 but not at P12 (compare Figure 2C and 2G). Therefore, we quantified both populations at P4, but only the "high" group at P12. Ki-67, which is only present when cells are actively proliferating [36,37], increased concordantly with the level of PDGF-Rα-GFP at P4 (Figure 2D, p < 0.05), but was inversely correlated at P12 (Figure 2H, p < 0.05). These data suggest that the varying PDGF-Rα expression levels are functionally significant with regards to proliferation at P4, as the proportion of proliferating fibroblasts within the PDGF-Rα-GFP+ group increases with increasing receptor expression levels. Conversely at P12, fewer of the PDGF-Rα-GFP+ fibroblasts are proliferating, and may mostly be in the G_0 _or G_1 _stages of the cell cycle.

### There is a positive correlation between fibroblast αSMA expression and proliferation at P4, but not at P12

**Figure 3:**
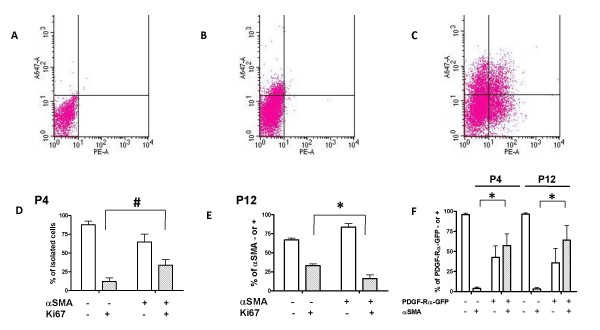
**The proliferative state of the myofibroblast (αSMA-expressing fibroblast) changes during alveolarization**. Representative FACS histograms from freshly isolated mouse lung fibroblasts (A-C). Fibroblasts were stained with phycoerythrin (PE)-α-smooth muscle actin antibody (x-axis) plus anti-Ki67 followed by a secondary antibody conjugated to Alexa Fluor 647 (A647, y-axis). Some samples were stained with only the A647 secondary antibody (A), non-immune IgG control for ki67 + secondary antibody and non-immune IgG control for α-smooth muscle actin (B), or with the anti-ki67 and anti-αSMA antibodies (C). Proportions of fibroblasts that stained positively or negatively for either of the antibodies was determined, averaged and summarized in column graphs for P4 (D) and P12 (E). The fibroblast populations represent a mixture of PDGF-Rα-GFP-positive and negative subpopulations. We determined that the majority of αSMA-containing fibroblasts expressed the PDGF-Rα at both P4 and P12 (F). The shaded bars represent the αSMA-containing fibroblasts. Background compensation was determined from the non-immune IgG controls prior to calculating the proportions of the different fibroblast populations. For the mice ages P4, n = 5 groups of 2-4 mice, and for P12, n = 7 groups of 2-4 mice. #, P < 0.05 and *, P < 0.001. For the FACS histograms, on the x-axis, "PE-A" = PE Area and on the y-axis, "A647-A" = A647 Area.

In order to define the correlation between the timing of fibroblast differentiation and proliferation during alveolar development, we compared proliferation of the αSMA-expressing myofibroblasts at the beginning (P4) and the middle (P12) of alveolar development. We analyzed the same flow cytometric output that was shown in Figure 2. The αSMA-containing myofibroblasts in this analysis represented a mixture of both PDGF-Rα-GFP+ and - populations. At P4, we observed that a greater proportion of αSMA-expressing myofibroblasts contained ki67 compared to the non-αSMA-expressing fibroblasts (Figure 3D, p < 0.05). In contrast at P12, the converse was observed, and a greater proportion of the fibroblasts that contained ki67 lacked αSMA (Figure 3E, p < 0.05). Relative to the αSMA non-expressing fibroblast population, our results demonstrate that a greater proportion of the αSMA-containing fibroblasts are proliferating at P4 than at P12. Because a greater proportion of αSMA-containing myofibroblasts was within the PDGF-Rα-GFP+ group at both ages, the data suggest that some of the proliferating myofibroblasts are PDGF-Rα-GFP+.

### Proliferating fibroblasts at P4 are more likely to express PDGF-Rα but not αSMA, and at P12, neither PDGF-Rα nor αSMA

**Figure 4:**
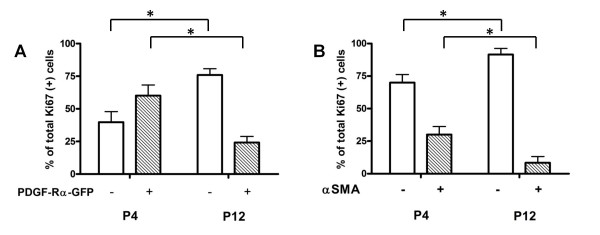
**Relative contributions of PDGF-Rα- and αSMA- expressing and non-expressing fibroblasts within the ki67+ population**. Column graphs summarizing in the ki67+ fibroblasts at P4 and P12, (A) the proportions of PDGF-Rα-GFP+ and - groups and (B) the proportions of αSMA + and - groups. The same flow cytometry output from Figures 2 and 3 was used for this analysis. The shaded bars represent ki67+ groups and the clear bars represent ki67- groups. *, p < 0.001.

The previous analyses (Figures 2 and 3) enabled us to examine age-related differences in the proportions of proliferating fibroblasts within the populations defined by either PDGF-Rα or αSMA expression as the primary variable. Using proliferation (Ki67) as the primary variable within the same data set, we analyzed either αSMA or PDGF-Rα as the secondary variable. Our results are summarized in Figure 4. We observed that at P4, the proliferating fibroblasts were mostly PDGF-Rα-GFP+ but αSMA- (Figure 4 A versus B, the left pair of columns, p < 0.05), but at P12, the proliferating fibroblasts were mostly PDGF-Rα-GFP- and αSMA- (Figure 4 A versus B, the right pair of columns, p < 0.05). These data suggest that, although some of the PDGF-Rα-expressing myofibroblasts are proliferating at P4, the majority of the proliferating PDGF-Rα-expressing fibroblasts are undifferentiated (lack αSMA). In contrast to P4, at P12 a majority of proliferating fibroblasts express neither PDGF-Rα nor αSMA, indicating a predominant proliferative effect of growth factors other than PDGFs at P12 [38].

### PDGF-Rα and αSMA expression levels positively correlate in developing alveolar tissues

Developmental studies using microscopy suggest that PDGF-Rα-expressing cells in the interstitium of the primary septae could be αSMA-expressing myofibroblast precursors [13,14]. However, the abundance of αSMA in PDGF-Rα-expressing cells in the developing secondary septa has not been studied. We hypothesized that if the PDGF-Rα-expressing mesenchymal cells are indeed myofibroblast precursors, then PDGF-Rα-expressing fibroblasts should also contain αSMA. We observed that freshly isolated total mouse lung fibroblasts expressing the PDGF-Rα were more likely to contain αSMA than their negative counterparts at both P4 and P12 (Figure 3F). However, flow cytometry did not enable us to examine tissue-colocalization of αSMA and PDGF-Rα-GFP.

**Figure 5:**
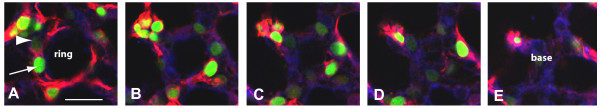
**Series of images representing a typical mature alveolus from a 12-day old PDGF-Rα-GFP mouse**. A representative 3 μm interval confocal z-stack of a single alveolus from the entry ring (A) to the base (E). The lung tissue was stained using Cy3-α-smooth muscle actin antibody (red) and the lipophilic carbocyanine dye, DiD (blue) for staining cell plasma membranes. The long white arrow shows a "high-expressing-PDGF-Rα " (high intensity of GFP) nucleus, and the white arrowhead shows a "low-expressing-PDGF-Rα-GFP" nucleus. The scale bar shown in (E) is 20 μm.

Therefore we examined αSMA abundance (pixel area density) proximate to "low" or "high" PDGF-Rα-GFP nuclei. In the PDGF-Rα-GFP mice a histone-nuclear localization sequence restricts GFP expression to the nucleus [30,32]. Instead of the continuous variable histogram developed by flow cytometric analysis, we used segmentation to develop a discrete variable ("low" or "high") that identified separate pixel intensity ranges for either "low" or "high" PDGF-Rα-GFP nuclei (Figure 5 arrow and arrowhead in A). We evaluated co-localization of αSMA and PDGF-Rα-GFP at the alveolar entry rings of different alveoli in tissue obtained from mice ages P4 and P12. A representative set of images for a single alveolus taken at 3 μm intervals through a confocal z stack is shown in Figure 5A-E.

**Figure 6:**
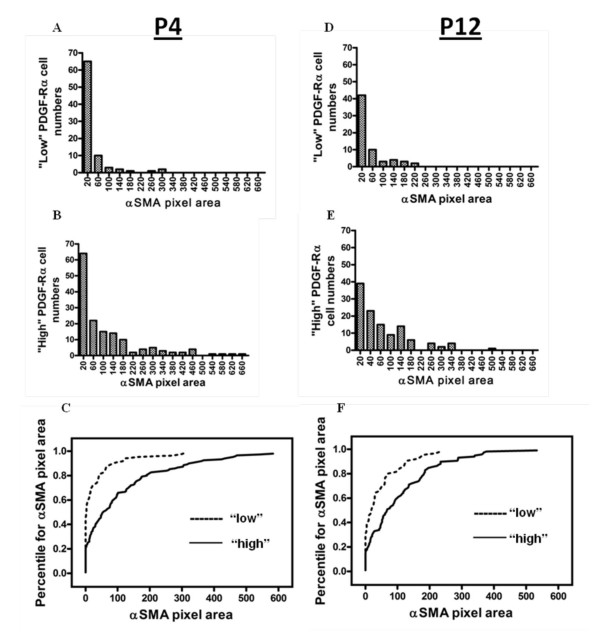
**Distribution patterns of αSMA around "low" and "high" PDGF-Ra-GFP nuclei in intact tissue**. Frequency distribution histograms summarizing confocal image analysis of stained intact tissue from mice aged P4 (A - C) and P12 (D - F). A circle measuring 9 μm in diameter was drawn around each PDGF-Rα-GFP nucleus in an alveolar entry ring (Figure 5), and the pixel area of αSMA that fell within this region of interest was calculated. Statistical differences between the distribution of αSMA within the "low" and "high" populations at each age were calculated using the Kolmogorov-Smirnov (KS) test. KS-test comparison percentile plots for P4 (C) and P12 (F) are shown. The αSMA pixel area values were ranked from lowest to highest, and the percentile for each value was determined (for example, the median value would be the 50^th ^percentile, or 0.5). The cumulative fractions were then plotted on the Y-axis, against the αSMA pixel areas on the X-axis. The αSMA distribution curves were statistically different (P < 0.05) between the "low" and "high" populations at each age. For each mouse, 16-18 alveolar entry rings were examined, and n = 3 mice at each age.

Using uniform segmentation criteria, pixels within a uniformly set range of Cy3 (αSMA) pixel intensities were selected. We then quantified the area of segmented αSMA pixels within a 4.5 μm radius around the center of each "low" and "high" PDGF-Rα-GFP nucleus at the alveolar entry ring. Our results are summarized in Figure 6. The frequency histograms show the numbers of cells of the "low" population at P4 (Figure 6A) and P12 (Figure 6D) and the "high" population at P4 (Figure 6B) and P12 (Figure 6E) associated with a particular αSMA pixel area binned range of values. We found that at both P4 and at P12, larger numbers of "high" than "low" PDGF-Rα-GFP nuclei were associated with progressively higher ranges of αSMA pixel area. In addition, we used the Kolmogorov-Smirnov nonparametric test to evaluate whether there was a statistical difference in the distribution of αSMA around the "low" and "high" PDGF-Rα-GFP nuclei at each age (Figure 6C and 6F, p < 0.05). The data suggest that the abundance of αSMA expression is related to the level of PDGF-Rα gene expression in adjacent alveolar cells.

### The "low" and "high" PDGF-Ra-expressing fibroblasts are similarly distributed at the alveolar entry ring and base

**Figure 7:**
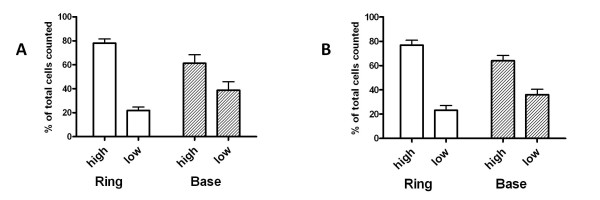
**PDGF-Rα expression patterns are similar at the alveolar entry ring and base**. The "low" and "high" PDGF-Rα subpopulations were enumerated at the alveolar entry ring and base, and the results are summarized in the column graphs representing numbers for the P4 (A) and P12 (B) animals. Each subpopulation is expressed as a percentage of the total numbers of cells at each respective level (ring or base). N = 3 mice for each age. For each mouse, at least 45 alveoli were examined.

We previously observed that a larger fraction of the alveolar cells that expressed PDGF-Rα localized to the base at P4 than at P12, but we did not distinguish cells based on PDGF-Rα expression levels [20]. We reasoned that PDGF-Rα expression levels could affect the responsiveness of the fibroblast to PDGFs, thus the distribution of the "low" and "high" populations at the ring versus the base could reflect the contributions of each population to secondary septal formation, and maintenance of alveolar structural integrity. We examined the relative abundance of "low" and "high" PDGF-Rα-expressing subpopulations at the entry ring (Figure 5A) versus the base (Figure 5E). At both the alveolar entry ring and base, "high" PDGF-Rα-expressing fibroblasts comprised a greater proportion of cells compared to the "low" population, and this pattern did not vary with age (Figure 7A and 7B). Thus there are no anatomical or temporal differences in the distribution of the "low" and "high" PDGF-Rα-expressing fibroblasts.

### PDGF-A/PDGF-Rα signaling suppresses α-smooth muscle actin expression in a neonatal mouse lung fibroblast cell line

Deletion of the *pdgfa *gene resulted in a loss of α-SMA-expressing myofibroblasts in the developing lung [13], clearly demonstrating the importance of PDGF-A/PDGF-Rα in myofibroblast ontogeny. However, it is still not known whether the effect of PDGF-A on αSMA expression is direct, or indirect. In an earlier study conducted by Dandre and Owens [39] using primary cultured smooth muscle cells, PDGF-BB suppressed αSMA only in sub-confluent but not confluent cells.

**Figure 8:**
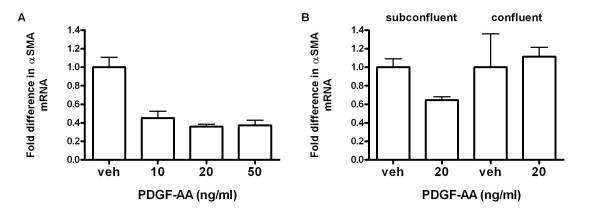
**PDGF-AA suppresses α-smooth muscle actin gene expression in sub-confluent mouse lung fibroblasts**. The neonatal mouse lung fibroblast cell line, Mlg, was stimulated with PDGF-AA (AA) for 24 h and levels of α-SMA mRNA for (A) increasing concentrations of PDGF-AA in sub-confluent cells (4.5 × 10^5 ^cells at the time AA was added), or (B) sub-confluent versus confluent cells (4.7 × 10^6 ^cells) was measured using real-time quantitative RT-PCR, with beta-2-microglobulin as the control "housekeeping" gene. For each reverse-transcribed RNA, 3 triplicate amplifications were performed. The error bars are 1 SD, columns are means of independent experiments. # = p < 0.05 compared to vehicle (veh).

To investigate the effects of PDGF-A on transdifferentiation, we exposed a continuous line of neonatal mouse lung fibroblasts (Mlg) to increasing doses of PDGF-AA for 24 h. PDGF-AA treatment resulted in suppression of αSMA mRNA in sub-confluent cells (Figure 8A, p < 0.05), suggesting that PDGF-A does not directly induce αSMA expression. In a separate experiment, we compared the effects of 20 ng/ml PDGF-AA on the steady state level of αSMA mRNA in sub confluent versus confluent cells. As shown in Figure 8B, the suppressive effects of PDGF-AA on αSMA expression are dependent on cell density.

### PDGF-AA/PDGF-Rα signaling represses the stimulatory effect of TGFβ on αSMA expression, and TGFβ releases αSMA from the PDGF-A-mediated suppressive effects

**Figure 9:**
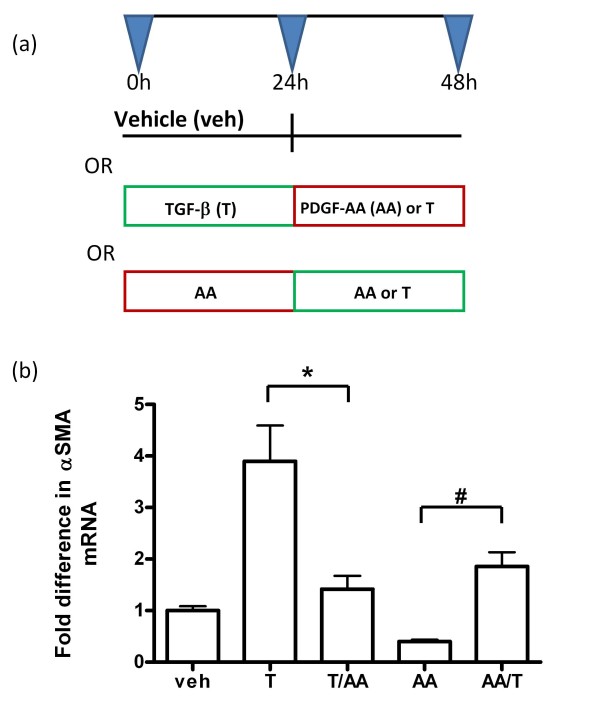
**PDGF-AA dampens the stimulatory effect of TGFβ on α-smooth muscle actin mRNA expression**. As illustrated in the schematic (A), sub-confluent Mlg cells were stimulated with either vehicle (veh), PDGF-AA (AA) or TGFβ (T) at the different time points. The levels of α-SMA mRNA were determined for the different treatment groups after a total of 48 h, using quantitative real time RT-PCR, with beta-2-microglobulin as the "housekeeping" gene. For each reverse-transcribed RNA, 3 triplicate amplifications were performed. The column graph in (B) represents the average of 5 independent experiments. The error bars represent standard deviations from the averages for each treatment group. *, P < 0.001 and #, P < 0.05.

TGFβ is well characterized as a positive regulator of αSMA expression in fibroblasts [22-24] and plays an important role in postnatal lung development [25-27]. Since PDGF-A had a repressive effect on αSMA in the subconfluent Mlg cells (Figure 8), we investigated whether PDGF-A suppresses TGFβ-mediated αSMA induction, and if TGFβ releases αSMA gene expression from the suppressive effects of PDGF-AA. Our results are summarized in Figure 9. We observed that TGFβ treatment alone stimulated α-SMA. However, addition of TGFβ followed by PDGF-AA resulted in significantly lower levels of α-SMA expression (p < 0.001). Conversely, addition of PDGF-AA alone resulted in a repression of αSMA, and addition of PDGF-AA followed by TGFβ resulted in a partial but significant recovery of αSMA expression (p < 0.05). These data demonstrate antagonistic interactions between intracellular signaling pathways activated by TGFβ and PDGF-A.

### PDGF-Rα expression does not preclude nuclear localization of SMAD 2/3 during alveolar development

**Figure 10:**
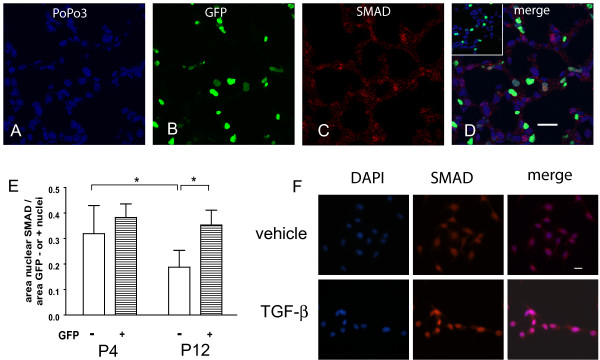
**PDGF-Rα expression does not preclude SMAD-dependent TGFβ signaling in the lung**. Lung tissue from PDGF-Rα-GFP mice aged P4 and P12 was stained for SMAD 2/3 followed by Alexa Fluor-568-conjugated secondary antibody (red, C) and PoPo 3 iodide nuclear counterstain (blue, A). The area of nuclear SMAD 2/3 was determined from merged images (D) in the nuclei cells that either express PDGF-Rα (yellow- red SMAD merged with green GFP) or do not express PDGF-Rα (magenta - red SMAD merged with blue pseudocolored PoPo3) cells. The inset in D (secondary antibody only) shows minimal levels of Alexa Fluor-568 staining in the absence of the primary antibody. Scale bars are 20 μm. Results are summarized in the column graph (E). The error bars represent standard deviations from the averages for each group. *, P < 0.001. To verify that nuclear translocation was TGFβ dependent, Mlg cells were treated with either vehicle or 0.125 ng/ml TGFβ (F) for 30 min and stained for SMAD 2/3 followed by 4', 6-diamidino-2-phenylindole (DAPI) nuclear counterstain. More SMAD 2/3 localized to the nuclei of TGFβ-treated cells.

Because our *in vitro *results suggested possible crosstalk between intracellular signaling pathways activated by PDGF-AA and TGFβ, we investigated TGFβ-dependent SMAD 2/3 signaling in the PDGF-Rα-expressing fibroblasts. At both postnatal days 4 and 12, we observed SMAD 2/3 nuclear localization in cells that were actively expressing the PDGF-Rα-GFP (Figure 10D). We then quantified the pixel areas occupied by SMAD 2/3 within the nuclei of either PDGF-Rα-expressing or non-expressing cells at both ages (Figure 10E). At P4, the pixel area of SMAD 2/3 was similar in the nuclei of both the PDGF-Rα-expressing and non-expressing cells. In contrast to P4, at P12 the nuclear SMAD 2/3 was maintained in PDGF-Rα-expressing but diminished in the non-expressing cells. These data suggest that PDGF-Rα gene expression does not preclude TGFβ/SMAD intracellular signaling in the intact lung.

To verify that nuclear localization of SMAD 2/3 depended on TGFβ-stimulation, we treated Mlg cells with either vehicle or TGFβ, and stained the cells using the same SMAD 2/3 antibody we used in the intact lung tissue. Compared to vehicle, there was increased nuclear SMAD 2/3 staining with TGFβ treatment (Figure 10F).

## Discussion

Although developmental studies [13,14] have clearly demonstrated that PDGF-A is required to expand and maintain alveolar myofibroblasts and septal outgrowth, the timing and mechanisms of PDGF-A action have not been defined. We have used GFP as a marker for endogenous PDGF-Rα gene expression to evaluate the proliferative and myofibroblastic characteristics of marked and unmarked cells during alveolar septal eruption (P4) and elongation (P12).

Our studies suggest that during septal eruption and elongation, the proliferative characteristics of PDGF-Rα-expressing fibroblasts vary with receptor expression levels. Based on fluorescence intensity, we observed differential PDGF-Rα expression in newly isolated fibroblasts using flow cytometry (Figure 1) and in intact tissue using fluorescence microscopy (Figure 5). This differential expression of the receptor is functionally significant, because "low" expressing fibroblasts are less proliferative than the "high" PDGF-Rα-expressing fibroblasts during septal eruption at P4 (Figure 2), and there are larger pixel area densities of αSMA protein proximate to the "high" than "low" PDGF-Rα-GFP nuclei at both P4 and P12 (Figure 6).

"Low" expressing fibroblasts may be less sensitive to PDGF-induced proliferation, either due to decreased available surface receptor expression, and/or decreased receptor activation. This hypothesis is consistent with previous reports of reductions in PDGF-A-mediated cell growth associated with diminished PDGF-Rα expression [40]. Hamilton et al [30] demonstrated using flow cytometry that PDGF-Rα expression overlapped with GFP fluorescence intensity in cells that had been derived from PDGF-Rα-GFP mouse embryos. Our flow cytometry data (Figure 1) also suggest that either the "low" population decreases as alveolarization progresses, or may become "high" PDGF-Rα-expressing. A proportional increase in "high" PDGF-Rα-expressing fibroblasts may be advantageous during septal elongation (P12) to support the formation of a mature, elastin-laden ECM. Further studies are required to determine whether "high" PDGF-Rα-expressing fibroblasts produce more elastin.

Our data also suggests that the overall proliferative state of the PDGF-Rα-expressing fibroblasts is different from the non-expressing fibroblasts, and may depend on their differentiative state. On postnatal day 4 (P4), at the onset of septal eruption, a larger proportion of fibroblasts within the PDGF-Rα- and αSMA- expressing groups were proliferative compared to the proportions within the non-expressing counterparts (Figures 2D and 3D). Conversely, at P12 these proportions of proliferating fibroblasts within the PDGF-Rα- and αSMA-expressing groups were smaller compared to the non-expressing counterparts (Figures 2H and 3E). A larger proportion of the PDGF-Rα-GFP positive fibroblasts were αSMA-positive compared to their PDGF-Rα-GFP negative counterparts at both P4 and P12 (Figure 3F). Of the proliferating fibroblasts, PDGF-Rα-expressing cells comprised a majority only at P4 and αSMA-containing cells comprised a minority at both P4 and P12 (Figure 4). These results are consistent with a transition of PDGF-Rα-expressing fibroblasts from a proliferative to a less proliferative more differentiated, ECM-producing state. A similar transition has been observed in fibrotic states where myofibroblasts initially proliferate at the site of injury, and thereafter become less proliferative and produce more ECM proteins [41]. Our results corroborate earlier electron micrographic studies showing that the ^3^H-thymidine labelling indices of fibroblasts in the rat and mouse lung was highest at P4, and afterwards declined [42]. Our results suggest that during septal eruption at P4, PDGF-Rα-expressing fibroblasts that are αSMA expressing and non-expressing proliferate more, potentially in response to PDGF-A. However, as alveolar septal elongation progresses and septal eruption is abating by P12, proliferation is less frequent among myofibroblasts and among PDGF-Rα expressing cells.

Although *pdgfa*-null mice lack αSMA-expressing myofibroblasts, it is not clear whether PDGF-A can directly stimulate αSMA expression in mouse lung fibroblasts. We thus stimulated a neonatal mouse lung fibroblast cell line with PDGF-AA and observed that PDGF-AA suppressed αSMA mRNA expression only in sub-confluent, but had no effect in confluent cells (Figure 8). These *in vitro *results do not necessarily contradict our *in vivo *observations of greater αSMA abundance around the "high" compared to the "low" nuclei (Figure 6). Differences in αSMA distribution around the "low" and "high" PDGF-Rα-expressing nuclei may reflect cells undergoing heterogenous differentiation pathways. PDGF-A/PDGF-Rα signalling during alveolar development may not directly induce the myofibroblast pathway, but instead regulate the numbers of fibroblasts entering the transdifferentiation pathway. This theory is consistent with earlier studies demonstrating that lung fibroblasts can switch between the myo- and lipo- fibroblast phenotype, depending on the *in vitro *or *in vivo *presence or absence of (i) lipofibroblast-promoting parathyroid hormone-related protein [43], or (ii) agonists of the nuclear lipogenic transcription factor, peroxisome proliferator-activated receptor gamma (PPARγ)[44,45]. Furthermore, studies in hepatic stellate cells (liver myofibroblasts) show that PDGFs inhibit lipofibroblast-promoting pathways [46], and the formation of adipose tissue [47]. It is possible that PDGF-A/PDGF-Rα signalling could suppress lipid storage thereby indirectly enhancing the myofibroblast phenotype during alveolar development. This hypothesis is supported by earlier studies conducted in our lab that showed a higher accumulation of lipid droplets in the "low" compared to the "high" PDGF-Rα-GFP nuclei [20].

Unlike PDGF-A, TGFβ promotes the myofibroblast phenotype *in vivo *and *in vitro*, and is also required for alveolar development. Therefore we also investigated whether PDGF-A and TGFβ have competing effects on αSMA expression in Mlg cells. We observed that TGFβ increased αSMA mRNA expression. However, subsequent addition of PDGF-AA, or treatment with PDGF-AA alone, reduced αSMA mRNA levels (Figure 9). Stimulation or repression of αSMA gene expression is complex, and involves the competitive binding of various positively- and negatively- acting cell-specific transcription factor complexes to multiple elements within the αSMA promoter-enhancer region, including the CC(AT-rich)6GG (CARG) and AGGAATG (MCAT) sequences, as well as the TGFβ 1 control element (TCE) [48-52].

The suppressive effects of PDGF-AA on αSMA mRNA expression are similar to observations made by Yoshida and colleagues [52], who found that the PDGF-BB repressed αSMA gene expression in smooth muscle cells by inducing the dissociation of the positive-acting myocardin related transcription factors (MRTFs or MKLs), from the CArG elements. This dissociation resulted from competitive binding between the PDGF-BB downstream effector, Elk1, and the MRTFs at earlier than at later timepoints. PDGF-AA may act through similar mechanisms to suppress TGFβ-mediated αSMA expression in fibroblasts, because PDGF-A/PDGF-Rα signaling also activates downstream Elk-1 [53] and MRTFs are expressed in both embryonic and adult mouse lung tissue [54]. Furthermore, TGFβ enhances the binding of positive-acting transcription factors such as serum response factor (SRF) to the CArG and TCE elements in fibroblasts [50].

Whereas in cultured Mlg cells, PDGF-AA antagonized TGFβ-mediated αSMA expression (Figure 9), nuclear SMAD 2/3 localization in PDGF-Rα-expressing cells was preserved in the intact tissue during both septal eruption (P4) and septal elongation (P12, Figure 10). However, in the non-expressing cells, nuclear SMAD 2/3 was lower at P12 compared to P4. Our results confirm earlier reports of persistent SMAD 3 and phospho-SMAD 2 staining in the developing alveolar interstitium until P28 [25]. To our knowledge, our findings are the first to address differences in SMAD 2/3 nuclear localization specifically in PDGF-Rα-expressing and non-expressing cells within the alveolar interstitium.

Our results also suggest that PDGF-Rα expression did not preclude TGFβ/SMAD signaling at the indicated stages of alveolar formation. We observed that a greater proportion of PDGF-Rα-expressing fibroblasts at both P4 and P12 contained αSMA compared to their non-expressing counterparts (Figure 3F). Therefore, other predominating factors may have maintained the myofibroblast phenotype, and overridden any *in vivo *suppressive effects of PDGF/PDGF-Rα signaling. Potential overriding factors include the development of focal contacts between the myofibroblasts and ECM proteins [55,56], integrin-mediated signaling via focal adhesion kinases [57], and increased mechanical stretching related to septal formation and respiration [58,59]. An alternative explanation could be that intracellular cross talk between PDGF-A and TGFβ signaling may occur via SMAD-independent pathways. The transcription factor known as early growth response-1 (Egr-1), which is induced by both PDGF-B [60] and TGFβ [61], is important for TGFβ-mediated collagen expression in mouse lung fibroblasts. This Egr-1-dependent activity is maintained even in fibroblasts derived from SMAD-3 null mice [61]. Interestingly, tumor necrosis factor alpha (TNFα) diminished TGFβ-mediated αSMA production in primary cultured human neonatal lung fibroblasts, via induction of Egr-1 without changing SMAD phosphorylation [62].

## Conclusion

Our study has provided valuable information concerning proliferative and differentiative characteristics of PDGF-Rα-expressing fibroblasts during alveolar development. We have demonstrated that PDGF-Rα expression levels *in vivo *positively correlate with αSMA protein expression during both P4 and P12, and with proliferation at P4, but not at P12. In addition, whereas the majority of proliferating fibroblasts at both stages are undifferentiated (non-αSMA-expressing), most of the fibroblasts are PDGF-Rα + at P4, but PDGF-Rα- at P12. In addition, we have demonstrated that PDGF-AA suppresses TGFβ-mediated αSMA induction *in vitro*, but PDGF-Rα expression does not preclude TGFβ-dependent nuclear localization of SMAD 2/3 *in vivo*. Therefore the data support an indirect mechanism for the PDGF-mediated regulation of the myofibroblast (αSMA) phenotype, and also imply that fibroblasts respond differently to PDGFs at different stages of alveolarization, possibly to regulate the timing of fibroblast differentiation and function. Furthermore, PDGF-A/PDGF-Rα signaling may separately or in combination with TGFβ, regulate myofibroblast differentiation and function during alveolar development. These interactions among the intracellular pathways may be SMAD-independent, and/or involve other myofibroblast-promoting factors such as mechanical tension and cell-ECM interactions.

Our results and those of others demonstrate the complex regulatory network that exists in the developing lung, and how the myofibroblast plays a key role in driving the process of alveolarization. Increasing understanding of the delicate molecular balance that regulates myofibroblast differentiation and function could lead to improved therapeutic strategies for fibrotic disease.

## List of abbreviations used

PDGF-A: Platelet-derived growth factor A; αSMA: Alpha-smooth muscle actin; PDGF-Rα: Platelet-derived growth factor receptor alpha; TGFβ: Transforming growth factor beta; GFP: Green fluorescent protein; SMAD: Homologs of the *drosophila *protein, Mothers Against Decapentaplegic (MAD) and the *C. elegans *protein, SMA; P4: postnatal day 4; ECM: Extracellular matrix; PBS: 0.145 M NaCl, 0.0015 M KH_2_PO_4_, 0.0027 M KCl, 0.0086 M Na_2_HPO_4_, pH 7.4; BSA: bovine serum albumin.

## Competing interests

The authors declare that they have no competing interests.

## Authors' contributions

PWK designed and conducted the cell culture, flow cytometry and SMAD 2/3 confocal experiments, analyzed and interpreted the data, and drafted the manuscript. AJH participated in manuscript editing and literature searches for technically relevant information, as well as provided technical advice about fibroblast isolations, RNA extractions and confocal microscopy. REG stained the intact mouse tissue with the αSMA antibody, collected the confocal images and contributed to editing of the manuscript. SEM was instrumental in the overall conception and direction of the study, advised in the interpretation and analysis of experimental data, and contributed substantially to the drafting and editing of the manuscript.
